# Engineering Amyloid-Like Assemblies from Unstructured Peptides via Site-Specific Lipid Conjugation

**DOI:** 10.1371/journal.pone.0105641

**Published:** 2014-09-10

**Authors:** María Pilar López Deber, David T. Hickman, Deepak Nand, Marc Baldus, Andrea Pfeifer, Andreas Muhs

**Affiliations:** 1 AC Immune SA, Lausanne, Switzerland; 2 Bijvoet Center for Biomolecular Research, Utrecht University, Utrecht, The Netherlands; Universität Erlangen-Nürnberg, Germany

## Abstract

Aggregation of amyloid beta (Aβ) into oligomers and fibrils is believed to play an important role in the development of Alzheimer’s disease (AD). To gain further insight into the principles of aggregation, we have investigated the induction of β-sheet secondary conformation from disordered native peptide sequences through lipidation, in 1–2% hexafluoroisopropanol (HFIP) in phosphate buffered saline (PBS). Several parameters, such as type and number of lipid chains, peptide sequence, peptide length and net charge, were explored keeping the ratio peptide/HFIP constant. The resulting lipoconjugates were characterized by several physico-chemical techniques: Circular Dichroism (CD), Attenuated Total Reflection InfraRed (ATR-IR), Thioflavin T (ThT) fluorescence, Dynamic Light Scattering (DLS), solid-state Nuclear Magnetic Resonance (ssNMR) spectroscopy and Electron Microscopy (EM). Our data demonstrate the generation of β-sheet aggregates from numerous unstructured peptides under physiological pH, independent of the amino acid sequence. The amphiphilicity pattern and hydrophobicity of the scaffold were found to be key factors for their assembly into amyloid-like structures.

## Introduction

Self-assembly of peptides and proteins into highly ordered structures is known to be determined by their primary sequence and promoted by external factors such as pH, organic solvents, metals and temperature. Rational *de novo* design of sequences that fold into a predicted complex structure under physiological conditions remains a challenging endeavor despite the progress made with the help of computational tools. Re-engineering a native scaffold is an alternative strategy that helps to gain knowledge of the folding principles and to identify amino acid subsets responsible for the adopted conformation. A common approach is the introduction of elements that promote or stabilize the folding of the peptide sequence such as templates, disulfide bridges, chelators or lipids. The introduction of hydrophobic moieties in short peptide sequences by lipidation has been shown to potentiate or stabilize secondary and tertiary protein-like structures. Depending on the number, position and type of fatty acids appended, diverse induced conformations (α-helix [1], [2], triple helix [1], [3], β-sheet/turns [4], [5]) were obtained for different peptide sequences. Sometimes, the presence of lipidic vesicles was required to promote folding of the modified sequence while the native peptide remained disordered in solution [1], [6], [7].

Aggregation of Aβ peptide into β-sheet assemblies (fibers, protofibrils, soluble oligomers, etc.) is one of the hallmarks of Alzheimer disease [8]. Aβ-amyloid fibers share several specific features with other fibers formed from unrelated peptides and proteins such as cross-β sheet quaternary structure, X-ray diffraction patterns, Congo Red-stained green birefringence and strong resistance to proteolytic degradation. Understanding the mechanism of aggregation is of great importance for the development of therapeutic and diagnostic agents. The hydrophobicity of the primary sequence seems to correlate with the amyloidogenic character of the amyloid species, as shown by the strong aggregation propensity of Aβ(1–42) compared to the less hydrophobic Aβ(1–40) [9], while the amphiphilic pattern may influence the fibril organization through maximization of hydrophobic contacts [10]–[14].

Based on these observations, we have envisaged the use of multi-lipidation to introduce hydrophobicity/amphiphilicity into short unstructured peptide sequences in order to control its final conformation and aggregation capability. The N-terminal part of Aβ(1–40/42) was chosen as a model sequence for the investigation since this region has been shown by NMR to be structurally disordered in the fibers [12], [15], [16]. Previously, we have shown that tetrapalmitoylated Aβ(1–15) (Palm1–15), when incorporated into liposomes, mimics the pathologic amyloid conformation and induces an immune response specific to aggregated Aβ species [17], [18]. In order to further improve our understanding of the basic driving forces in amyloid-like assembly, we have characterized in-depth the peptide Palm1–15 without the liposomal surface influence by several physico-chemical techniques (CD, ATR-IR, ThT fluorescence, DLS, ssNMR and EM). In addition, we have investigated the influence of different parameters (type and number of lipid chains, peptide sequence, peptide length and net charge) upon the adopted conformation and aggregation state of a range of other peptides. Due to the highly hydrophobic nature and non-aqueous solubility of the lipopeptides, HFIP was used as co-solvent at low percentage (1–2%, v/v) and at a constant ratio peptide/HFIP to minimize its influence on the fibrillization process and thus to isolate the effect of the studied parameters. Although the co-solvent may potentially play a role in the aggregation process of the peptides, the scope of this work did not include the study of the effect of increasing HFIP concentration on the morphology and stabilization of assemblies. Specifically, this work presents direct evidence for the control of β-sheet conformation by site-specific multi-lipidation of unstructured peptide sequences in nearly aqueous conditions.

## Results

### Scaffold design and characterization

**Figure 1:**
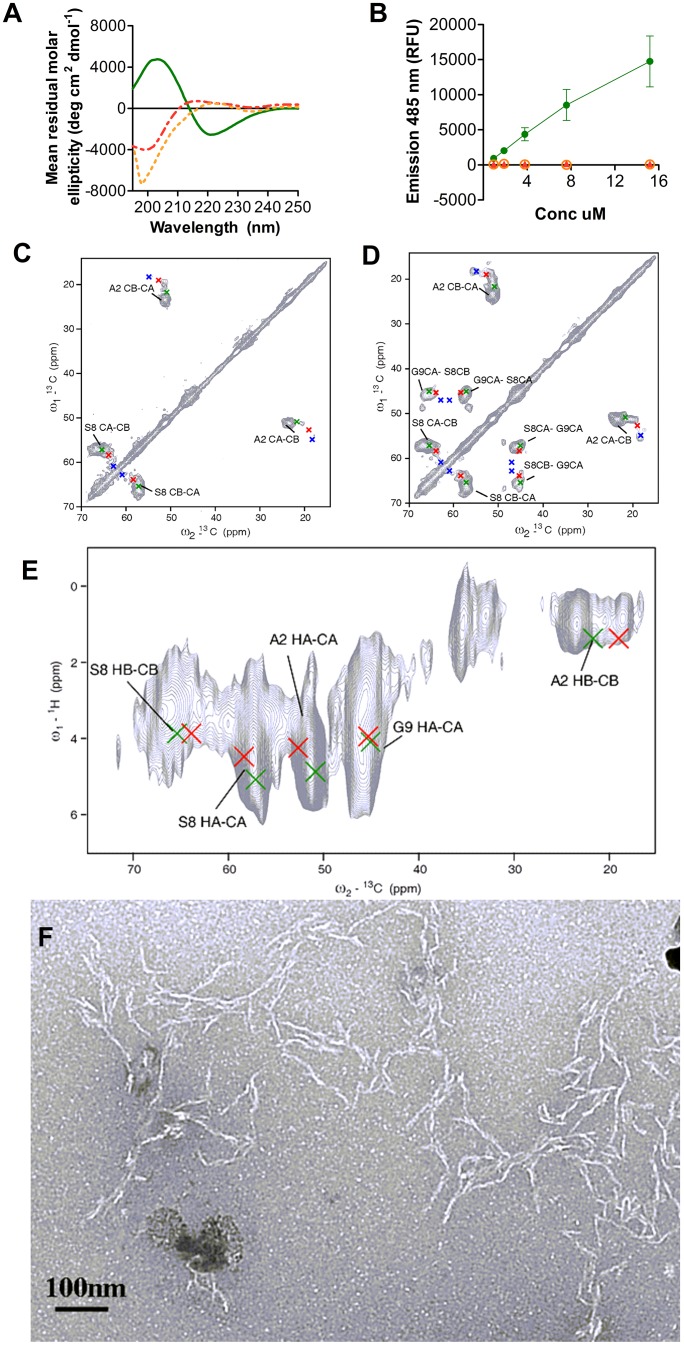
Characterization of β-sheet amyloid-like aggregates of Palm1–15. A) CD spectra at 30 µM peptide Palm1–15 (green) and controls Acetyl1–15 (red) and Aβ1–15 (orange) in 2% HFIP/PBS (v/v). B) Profile of ThT emission with different peptide concentrations in the presence of 24 µM of dye. Fluorescence was measured at 485 nm with excitation at 440 nm. C, D, E) ssNMR of Palm1–15 uniformly labeled at Ala2, Ser8 and Gly9: (^13^C-^13^C) 2D PDSD correlation spectra with mixing times of 20 ms (C) and 150 ms (D); ^1^H-^13^C FSLG-HETCOR spectra (E) together with chemical shift predictions based on secondary structure [38] (red for random coil, green for β-sheet, blue for α-helix). F) Electron micrographs of negatively stained Palm1–15 aggregates formed over 24 hour incubation in 2% HFIP/PBS (v/v). Samples were negatively stained with 2% Uranyl Acetate in water. Scale bar 0.1 µm. Magnification 22000 x.

As previously reported [17], Aβ(1–15) peptide was decorated at both N- and C-termini with two lysine residues acylated on their side-chains with palmitic acid to give model sequence Palm1–15 (Group 1 in Table 1). The scaffold displayed a β-sheet conformation in 2% hexafluoroisopropanol (HFIP, v/v) in PBS solution and formed soluble β-sheet aggregates, as shown respectively by CD and ThT fluorescence (Figure 1A, B). In contrast, the unstructured native sequence Aβ(1–15) and control tetraacetylated peptide Acetyl1–15 (Group 1 in Table 1) were random coil in solution as evidenced by the background ThT signal (Figure 1A, B).

**Table 1:** Peptide sequences of lipopeptides and controls.

Group	Name	Peptide	Acyl group
**1**	Palm1–15	H-K(X)-K(X)-DAEFRHDSGYEVHHQ-K(X)-K(X)-OH	X = Palmitoyl
	Acetyl1–15		X = Acetyl
**2**	Palm15-1	H-K(X)-K(X)-QHHVEYGSDHRFEAD-K(X)-K(X)-OH	X = Palmitoyl
	Acetyl15-1		X = Acetyl
**3**	scPalm15	H-K(X)-K(X)-GHEAYHSVERFDDQH-K(X)-K(X)-OH	X = Palmitoyl
	scAcetyl15		X = Acetyl
**4**	Palm1–9	H-K(X)-K(X)-DAEFRHDSG-K(X)-K(X)-OH	X = Palmitoyl
	Acetyl1–9		X = Acetyl
**5**	Palm1–5	H-K(X)-K(X)-DAEFR-K(X)-K(X)-OH	X = Palmitoyl
	Acetyl1–5		X = Acetyl
**6**	Palm1–15(D7K)	H-K(X)-K(X)-DAEFRHKSGYEVHHQ-K(X)-K(X)-OH	X = Palmitoyl
	Acetyl1–15(D7K)		X = Acetyl
**7**	Palm1–15(E3A, D7K)	H-K(X)-K(X)-DAAFRHKSGYEVHHQ-K(X)-K(X)-OH	X = Palmitoyl
	Acetyl1–15(E3A, D7K)		X = Acetyl
**8**	Palm1–15(E3K, D7K)	H-K(X)-K(X)-DAKFRHKSGYEVHHQ-K(X)-K(X)-OH	X = Palmitoyl
	Acetyl1–15(E3K, D7K)		X = Acetyl
**9**	Palm1–15(E3K, D7K,E11K)	H-K(X)-K(X)-DAKFRHKSGYKVHHQ-K(X)-K(X)-OH	X = Palmitoyl
	Acetyl1–15(E3K, D7K,E11K)		X = Acetyl
**10**	Palm1–15(4C)	H-DAEFRHDSGYEVHHQ-K(X)-K(X)-K(X)-K(X)-OH	X = Palmitoyl
	Acetyl1–15(4C)		X = Acetyl
**11**	Palm1–15(2C)	H-DAEFRHDSGYEVHHQ-K(X)-K(X)-OH	X = Palmitoyl
	Acetyl1–15(2C)		X = Acetyl
**12**	Palm1–15(1N1C)	H-K(X)-DAEFRHDSGYEVHHQ-K(X)-OH	X = Palmitoyl
	Acetyl1–15(1N1C)		X = Acetyl
**13**	Palm1–15(1C)	H-DAEFRHDSGYEVHHQ-K(X)-OH	X = Palmitoyl
	Acetyl1–15(1C)		X = Acetyl
**14**	Dodecyl1–15	H-K(X)-K(X)-DAEFRHDSGYEVHHQ-K(X)-K(X)-OH	X = Dodecanoyl
	Octyl1–15		X = Octanoyl
	Butyl1–15		X = Butanoyl

A complementary structural analysis of Palm1–15 was performed by Magic-Angle Spinning (MAS) ssNMR, using the lyophilized crude powder. Two-dimensional (^1^H, ^13^C) and (^13^C, ^13^C) NMR correlation experiments were conducted on Palm1–15 site-specifically uniformly (^13^C, ^15^N)-labeled at residues Ala2, Ser8 and Gly9 (Figure 1C, D, E). The comparison between the experimental secondary chemical shifts identified in the spectra with predictions suggested that Palm1–15 predominantly adopts a β-strand conformation (Table S1). From 2D integration of the resolved carbon cross peaks in the ^13^C-^13^C ssNMR correlation spectra using Topspin 3.0, Bruker Biospin, it was estimated that Palm1–15 has more than 75% β-sheet content. The amyloid-like β-sheet structure of scaffold Palm1–15 was further confirmed by ATR-IR spectroscopy by the presence of the characteristic amide band I with maximum around 1620–1640 cm^−1^. The minimum displayed at 1625 cm^−1^ in the second derivative of the absorbance spectra of Palm1–15 aggregates was clearly distinct from the minimum at 1648 cm^−1^ of Acetyl1–15 amide band I, typical of a disordered structure (Figure S1). In addition, for Palm1–15 a weak component at 1695 cm^−1^ could indicate an antiparallel arrangement of β-strands, as in pre-fibrillar oligomers [19]. The morphology of Palm1–15 soluble aggregates was examined by Electron Microscopy after 24 h incubation (Figure 1F). Abundant unbranched coiled fibrils were present, although bundles and exfoliated sheet regions were also observed. Measured isolated fiber lengths from 40–90 nm (average length 57 nm±12 nm, Table S2) with 6 nm average thickness (Table S3) were found to be in the range of those observed for amyloid fibrils and protofibrils [20]. On the other hand, no ordered structures were detected in the samples of Acetyl1–15 and native Aβ(1–15) when analyzed by negative staining EM and with the same protocol (Figure S2).

Analysis of Palm1–15 aggregates by DLS revealed an average hydrodynamic diameter *D_h_* of ∼350 nm with high size homogeneity (polydispersity index, pDI = 0.20) at 15 µM of peptide (Figure S3). Translation of hydrodynamic diameter into fibril length was calculated assuming a rigid cylindrical rod structure as described (see Method S1) [21]. Based on the EM data this is a rough approximation due to the presence of both unbranched and associated structures. From a fibril diameter of 6 nm, a fibril length of approximately 2 µm is calculated, which is considerably higher than that found by EM. This discrepancy may be due to fibril instability during EM sample preparation. Indeed, serial dilution of this stock solution with 1% HFIP in PBS (v/v) followed by immediate DLS measurements revealed much smaller particles, with a logarithmic relationship between peptide concentration and hydrodynamic diameter, presumably due to a shift in the monomer-fibril equilibrium. Nevertheless, significant sized aggregates (measured hydrodynamic diameter *D_h_* 155 nm, calculated length ∼500 nm) were still found even at low peptide concentration (0.25 µg/mL). Since the concentration of HFIP was kept constant, it is assumed that the observed peaks correspond to peptide aggregates or peptide co-adducts with HFIP, and are not due to HFIP alone.

The thermal folding of Palm1–15 fibers was examined by CD using as co-solvent the synthetic detergent β-OG instead of HFIP given the high volatility of the latter (boiling point, 58.2°C) (Figure S4). The change in ellipticity at 221 nm and 205 nm was monitored from 25°C to 95°C in steps of 5°C. The minimum at 221 nm disappeared with increasing temperature, reflecting the shift from a β-sheet conformation to a disordered state as indicated also by the decrease in intensity of the maximum at 205 nm. The presence of an isodichroic point at 215 nm could indicate a two-state equilibrium between random coil and β-sheet species during the unfolding process. The melting temperature, roughly calculated from the first derivative of the fitting curve, was found to be ∼64°C.

### Influence of peptide length and sequence

**Figure 2:**
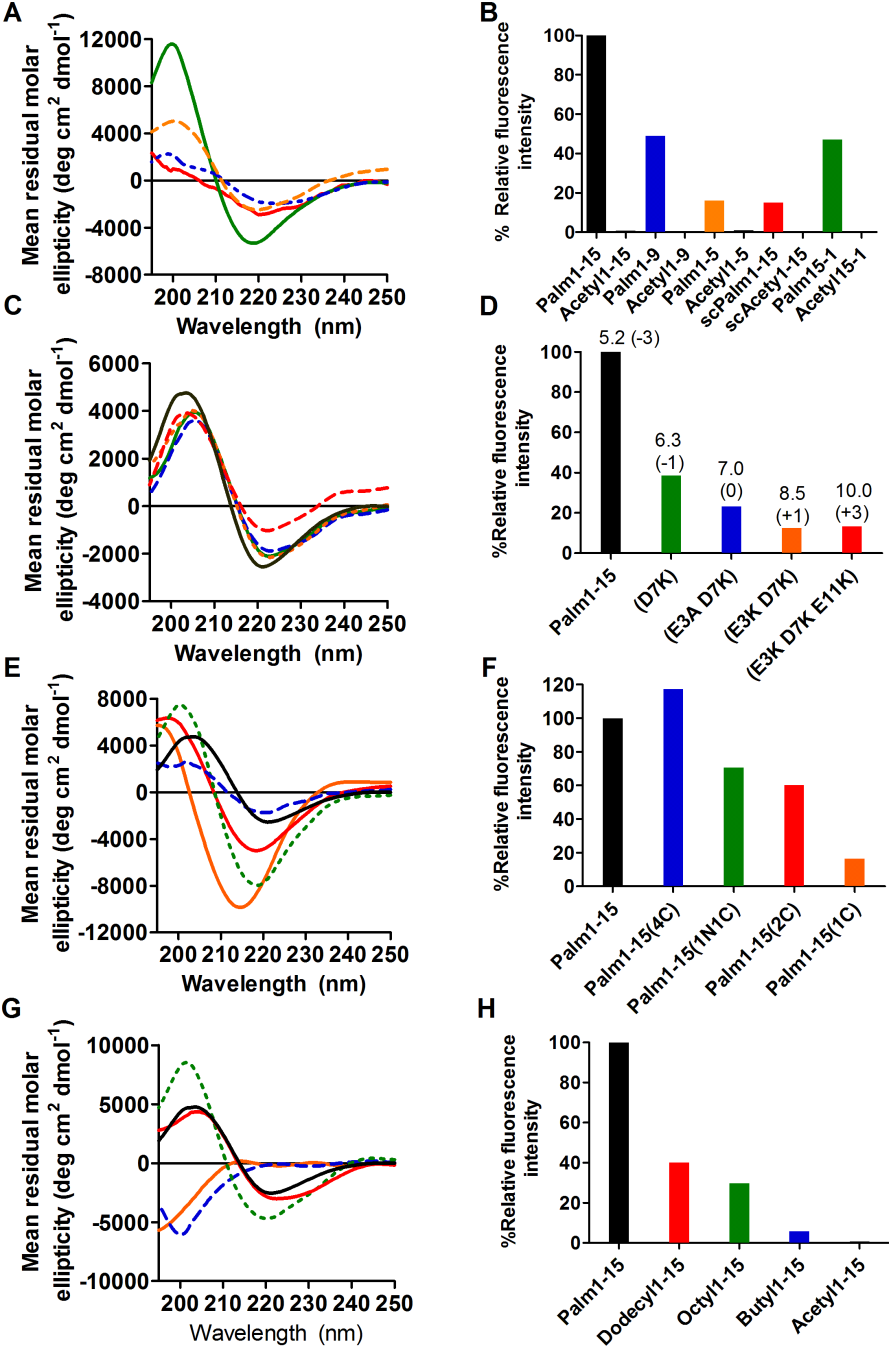
Parameters influencing scaffold conformation. A) CD spectra and B) ThT fluorescence of tetrapalmitoylated peptides with shortened length, 9 or 5 amino acids (Palm1–9 in blue and Palm1–5 in orange respectively) or different order of amino acids, reverse or scrambled (Palm15-1 in green and scPalm15 in red, respectively) to model sequence Palm1–15. C) CD and D) ThT of tetrapalmitoylated peptides with different isoelectric point with pI (and net charge) value indicated on top of each column (Palm1–15(D7K) in green, Palm1–15(E3A, D7K) in blue, Palm1–15(E3K, D7K) in orange, Palm1–15(E3K, D7K, E11K) in red and Palm1–15 in black). E) CD and F) ThT of peptides with different number/position of palmitic chains (Palm1–15(1C) in orange, Palm1–15(2C) in red, Palm1–15(1N1C) in green, Palm1–15(4C) in blue and Palm1–15 in black). G) CD and H) ThT of peptides acylated with different lipid chain length (Acetyl1–15 in orange, Butyl1–15 in blue, Octyl1–15 in green, Dodecyl1–15 in red and Palm1–15 in black). Peptides were 30 µM in 2% HFIP/PBS (v/v) (CD) and 15 µM in 1% HFIP/PBS (v/v) (ThT, 24 µM). Fluorescence was measured at 485 nm with excitation at 440 nm. Values are the average of 3 replicates, normalized to the Palm1–15 emission (taken as 100%).

In order to probe the sequence generality of the scaffold-induced amyloid conformation, Palm1–15 derived lipidated sequences with shortened peptide length or with different order of amino acids were prepared and analyzed by CD and ThT fluorescence (Figure 2A, B). Shortened sequences Palm1–9 and Palm1–5 (with 9 and 5 residues respectively, Group 4 and 5 in Table 1) also adopted a β-sheet structure in solution like model Palm1–15, as shown by the minimum around 220 nm in the CD spectra (Figure 2A). Similar CD profiles were obtained for the reverse sequence Palm15-1 and a scrambled sequence scPalm15 (Group 2 and 3 in Table 1), showing that the order of amino acids did not greatly influence the resulting secondary conformation (Figure 2A). In contrast, the corresponding control sequences bearing acetylated lysines displayed a random coil conformation in solution (Figure S5). As with model Palm1–15, these Aβ-derived palmitoylated sequences showed the positive signal in the ThT assay characteristic of amyloid-aggregates while only a background signal was obtained with the corresponding acetylated peptides (Group 2, 3, 4 and 5 in Table 1, Figure 2B).

### Influence of peptide net charge

The influence of the peptide net charge in the conformation adopted by the lipoconjugates was probed with mutant sequences. Non-conservative mutations were introduced at several positions of the Aβ1–15 sequence in order to obtain differently charged peptide scaffolds. While the Palm1–15 model sequence has an isoelectric point (pI) of 5.2 (net charge of −3) the substitution of some glutamic or aspartic residues by lysine or alanine yielded Palm1–15(D7K), Palm1–15(E3A, D7K), Palm1–15(E3K, D7K) and Palm1–15(E3K, D7K, E11K) with pIs of 6.3, 7.0, 8.5 and 10.0 respectively (with net charge of −1, 0, +1 and +3 respectively, Group 6, 7, 8 and 9 in Table 1). In conclusion, the CD spectra suggest that, independently of the net charge, all mutated peptides adopt a β-sheet conformation (Figure 2C). In addition, all mutated scaffolds were positive in ThT assay indicating an amyloid-like structure in solution similar to model Palm1–15 (Figure 2D). In contrast, acetylated mutated sequences were disordered with no aggregation tendency as observed by CD and ThT (Group 6, 7, 8 and 9 in Table 1, Figure S6).

### Influence of the position and number of palmitic chains

The effect of the lipidation pattern on the propensity of the unordered Aβ(1–15) peptide to adopt a β-sheet conformation was then studied. The model sequence was decorated with different numbers of palmitic chains at the N- and/or C-terminus: Palm1–15(4C), Palm1–15(2C) and Palm1–15(1C) with 4, 2 or 1 palmitic chains at the C-terminus and Pal1–15(1N1C) with one palmitic chain at both peptide ends (Group 10, 11, 12 and 13 in Table 1). In general, increasing the number of lipid chains induced more β-sheet conformation by CD (Figure 2E). Peptide β-sheet aggregation was also modulated by the lipidation pattern as shown by the intensity of the ThT fluorescence signal: increased hydrophobicity was paralleled by a stronger ThT signal, reflecting a higher aggregation state (Figure 2F). The acetylated version of each lipoconjugate showed a random coil conformation by CD and background ThT signal (Group 10, 11, 12 and 13 in Table 1 and Figure S7). The different aggregates induced by the distinct lipidation patterns were further characterized by ATR-IR. All the peptides showed a broad amide band I with maxima in the β-sheet region from 1620 to 1640 cm^−1^ (Figure S8). A quantitative analysis of the amide I band for each peptide was done by curve-fitting to estimate the percentage of secondary structural components (Table 2 and Figure S8). In all cases, the major component of the amide band I was assigned to β-sheet structure with the exception of Palm1–15(1C) and control Acetyl1–15 which showed higher and mainly random coil contributions respectively, in agreement with the CD results. In addition, Palm1–15 and Palm1–15(1N1C) showed the strongest component band around 1690–1696 cm^−1^, indicative of antiparallel β-sheet organization.

**Table 2:** Quantitative analysis of ATR-FTIR spectra of the peptides with different lipidation pattern.

	Possible structure assignment of ATR-IR band (cm^−1^)									
	β-sheet		Random coil		α-helix/loop		Turn		β-sheet	
	cm^−1^	(%)	cm^−1^	(%)	cm^−1^	(%)	cm^−1^	(%)	cm^−1^	(%)
**Palm1–15**	1603/1625	47	1644	29			1666	23	1690	9
**Palm1–15(4C)**	1617/1628	35	1639	26	1659	36	1689	2		
**Palm1–15(1N1C)**	1626	58	1648	25			1674	13	1694	5
**Palm1–15(2C)**	1627	47	1649	27			1673	22	1694	3
**Palm1–15(1C)**	1622	38	1649	35			1673/1689	24	1696	3
**Acetyl1–15**	1624/1634	19	1649	47			1671/1689	34		

### Influence of lipid chain length

Palm1–15 scaffold was modified by shortening the lipidic chains of the terminal lysines in order to determine the acyl length threshold (Group 14 in Table 1). Lipidation with fatty acids of decreasing lengths (16, 12, 8 and 4 carbon atoms) showed only a clear reduction of the β-sheet conformation for the C4 acylated sequence as shown by CD (Figure 2G). Importantly, Butyl1–15 showed a disordered structure similar to the tetraacetylated control sequence. Interestingly, a decreasing trend in β-sheet aggregation was observed by ThT assay, where the intensity of the signal decreased with the length of the lipid chain (Figure 2H).

## Discussion

Lipidation of peptide sequences provides a specific way to modulate native peptide structure through association via hydrophobic interactions. Here we describe a methodology for inducing a β-sheet conformation into native disordered sequences through lipidation at both peptide termini. Palmitoylation of the native disordered Aβ(1–15) sequence provided the basic scaffold characterized by a β-sheet structure based on CD and IR. Moreover the aggregation state of the peptide resembled the cross-β-sheet structure of fibers based on EM and ThT fluorescence. In contrast, the control scaffold bearing acetyl groups instead of palmitoyl chains, as well as the non-lipidated native sequence, lacked order as revealed by CD, IR, EM and ThT. These assays were measured with a solution of peptide in 1–2% HFIP in PBS (v/v) to help solubilize the hydrophobic peptides while keeping mostly aqueous conditions. Fluorinated solvents (trifluoroethanol (TFE), HFIP) at high concentrations are common solubilizing agents for hydrophobic proteins and peptides, that promote intramolecular hydrogen bonds, stabilizing the secondary structures such as α-helix, turns and beta-hairpins [22]–[25]. On the contrary, when used as cosolvent at low concentration (1–4%) in aqueous solutions, HFIP (as well as TFE at <20%) has been shown to promote fibrillization through enhancement of hydrophobic interactions between solutes at the interface of the formed HFIP microdroplets [26]–[28]. In our case, the diluted polar solvent probably catalyzes the aggregation of the poorly water-soluble Palm1–15 by concentrating the amphiphilic monomer at the interface between the aqueous and non-polar phases. The higher water solubility and the lack of fatty acid chains of Acetyl1–15 and Aβ(1–15) peptides would diminish their tendency to interact with the interface and favor them to remain free in aqueous solution, adopting the disordered native conformation.

The ssNMR secondary chemical shift analysis of the site-specifically uniformly (^13^C, ^15^N)-labeled Palm1–15 peptide (Ala2, Ser8 and Gly9) suggests a dominant presence of β-sheet content (>75%) along with a certain degree of structural heterogeneity indicating amyloid polymorphism at the molecular level. The resulting ^13^C and ^1^H line widths measured in this lipopeptide compared favorably to previous results obtained in amyloid peptides such as polyglutamine fibrils [29], suggesting that the spectral quality in these preparations is comparable to fibrils that have been shown to fold into β-sheet structures. Indication of an anti-parallel organization of the β-sheets inferred from the IR profile could not be confirmed by ssNMR due to the labeling pattern of Palm1–15 (Ala2, Ser8 and Gly9). The inter-strand topology (parallel vs. anti-parallel) could, for example, be explored in future experiments on fibrils obtained using mixed peptides that are N- or C-terminally ^13^C, ^15^N labeled. Anti-parallel strand topologies would then give rise to through-space ssNMR correlations between N- and C-terminal residues in, for example, (^13^C, ^13^C) correlation experiments.

The soluble HFIP-induced Palm1–15 aggregates appeared in EM as short, curly and unbranched fibers with 6 nm diameter and 57 nm average length. The morphology and dimensions of these structures were closer to the ones characteristic of protofibrils (short flexible fibrils of 4–10 nm in diameter and up to 200 nm in length) rather than mature fibers. Disaggregation of unstable fibers during EM sample preparation may explain why longer fiber lengths (up to 2 µm) were calculated from size measurements by DLS. Upon dilution, aggregate size was rapidly reduced, even too fast to be monitored in real-time. Thus HFIP-promoted aggregation is reversible and concentration-dependent. The fiber instability was further confirmed by the estimated low melting temperature of similar Palm1–15 β-sheet aggregates generated at the Octyl β-D-glucopyranoside (β-OG)/PBS amphipathic interface. With increasing temperature, a shift from a β-sheet conformation to a disordered state was induced, contrary to the β-sheet further folding and oligomerization observed for Aβ [30]. Random coil monomers are in dynamic equilibrium with the β-sheet protofibrils as indicated by the isodichroic point at 215 nm observed during CD denaturation experiments. Although the nature of the interface (β-OG, HFIP, etc) could have an effect on the morphology and stability of the fibers, a certain similarity among the induced β-sheet aggregates is expected based on the intrinsic structural properties of the lipopeptide. Unstable β-sheet aggregates of Aβ peptide, similar to those described here, have also been reported for Aβ(1–40) aggregates formed in aqueous solution at the interface with hydrophobic solvents, including HFIP [26] and chloroform [31] or with detergents such as SDS [32]. This is also consistent with our finding of aggregation of Palm1–15 within liposomal membranes [17] and suggests that other hydrophobic surfaces such as lipoproteins or synthetic polymers may also promote aggregate assembly/disassembly.

The scaffold-induced β-sheet aggregation of the native disordered peptide was found to be independent of the primary sequence as shortening the peptide length, altering the amino acid order or introducing mutations, resulted in similar structures as shown by CD, although with different extent of aggregation. Importantly, amyloid-like assemblies were obtained in all cases under close to physiological conditions, without the need of stress factors (pH, temperature, etc). The intrinsic hydrophobicity of the scaffold was enough to promote the association of the lipopeptides into fibers.

The influence of the amphiphilic pattern on the fibrillization tendency of the scaffold was then investigated by changing the number and position of the fatty acid chains. Both tetrapalmitoylated constructs Palm1–15 and Palm1–15(4C) strongly aggregated into β-sheet structures. The different arrangement of the palmitoyl chains seems to have an impact on the organization of the fibers as evindenced by EM: Palm1–15(4C) showed mostly bundles of fibers (Figure S2) in contrast with the unbranched separate fibers of Palm1–15. Likewise, positioning of two fatty acid chains at the C-terminus or one at each ends of the peptide sequence resulted in similar β-sheet structures although the dipalmitoylated peptides showed lower aggregation state than their more acylated counterparts, as expected from the corresponding decrease in hydrophobicity. Finally, removal of one palmitoyl chain in Palm1–15(2C) to give the Palm1–15(1C) resulted in an increase of the random coil contribution and lower aggregation of the peptide. The amphiphilic pattern of Palm1–15(2C) seems to mimic the one of Aβ(1–42) as the shared hydrophilic region (1–15) is followed in both cases by a hydrophobic fragment: the amino acids 29–42 in Aβ(1–42) or the fatty acid chains in the scaffold. Reducing the hydrophobic part of Palm1–15(2C) results in a decrease in the fibrillization propensity as observed when shortening the C-terminal region from Aβ(1–42) into the less amyloidogenic Aβ(1–40).

An alternative strategy to decrease the hydrophobicity of the scaffold in order to investigate the effect on its β-sheet aggregation capability consisted of acylating the four lysine terminal residues with shorter lipid chains of 4, 8 or 12 carbon atoms. Dodecyl1–15 and Octyl1–15 maintained the β-sheet conformation by CD while Butyl1–15 displayed a disordered structure comparable to the one of Acetyl1–15. The fibrillization propensity of the lipoconjugate diminished proportionally with the length of the alkyl tail as revealed by the decrease in the intensity of the ThT signal. Interestingly, the total number of carbon atoms did not fully correlate with the aggregation tendency of the scaffold as dipalmitoylated and monopalmitoylated Aβ(1–15) (32C and 16C in total, respectively) showed stronger ThT signal than Octyl1–15 and Butyl1–15 with correspondingly equal number of carbon atoms. Partitioning of lipopeptides into membranes is known to be favored by the increasing length of alkyl tail (>10C) and number (two hydrocarbon chain anchors better than one) [33]. Consistently, the association of these lipopeptides on the HFIP interface seems to be promoted proportionally by multi-lipidation (>1 chain) with fatty acid chains of lengths ≥8C and this enhancement correlates to the extent of peptide-amphiphile β-sheet aggregation. This mechanism would be in line with the observation that the amphiphilic Aβ peptide associates with cell membranes, specifically with the lipid-raft markers cholesterol and ganglioside GM1, and this accumulation would promote its oligomerization into neurotoxic species [34]. Moreover, modification of Aβ through covalent-linking with reactive lipid-aldehydes as 4-HNE or oxysterols (metabolites generated by oxidative stress in AD) has been shown to increase its hydrophobicity and propensity to misfold into pathological quaternary structures [35]. Hence the metabolite-modified Aβ could act as a seed at the cell surface, lowering the critical concentration of the unmodified Aβ and accelerating its amyloidogenesis.

## Conclusion

In summary, this study presents the sequence-independent generation of amyloid-like assemblies from unstructured peptides through site-specific lipidation, under mild, close to physiological conditions and without the requirement for the standard effectors such as pH, ionic strength, enzymes, temperature or light. The presence of HFIP as co-solvent helps to solubilize the hydrophobic peptides and may facilitate the formation of the amyloid-like aggregates. Hydrophobicity and amphiphilic pattern were found to be key factors governing the self-assembly of the amyloid β-derived constructs in the presence of a constant low percentage of HFIP. Characterization of the ordered structures generated from these model lipopeptides contributes to the general understanding of the mechanism of amyloid-like formation. In addition, the amphipathic scaffolds described herein could find widespread application as immunogenic constructs, peptidomimetics, bioactive nanomaterials or antimicrobial agents.

## Materials and Methods

Peptides were synthesized by solid-phase synthesis and characterized by HPLC and MALDI as previously described [17].

### CD spectroscopy

CD spectra were acquired on a Jasco-815 spectropolarimeter (JASCO International Co. Ltd., Japan) with a 0.1 cm path length quartz cuvette at 23°C. Measurements were made with a 1.0 nm bandwidth and 0.5 nm resolution. A scan speed of 50 nm/min was employed with response time of 8 s. Solvent control spectra (4 scans) were averaged and subtracted from the average of 8 scans of each sample spectrum. All spectra ([θ]_obs_, degrees) were smoothed before being converted to mean residue molar ellipticity ([θ], degrees cm^2^ dmol^−1^) with the equation [θ] = [θ]_obs_×(MRW/10*lc*), where MRW is the mean residue molecular weight (MW/number of residues), *l* is the optical path length (cm) and *c* is the concentration (g/cm^3^). For CD analysis, peptides were dissolved in neat HFIP at 1.50 mM concentration and diluted (1∶100) or (2∶100) with PBS to give final peptide concentrations of 15 and 30 µM respectively (in 1% or 2% HFIP/PBS, v/v respectively). The ratio HFIP/peptide ratio was kept constant and both conditions (1% or 2% HFIP) were expected to be equivalent due to the low % of co-solvent. The results of 2% HFIP were selected as they were qualitatively similar but gave better signal to noise ratio than 1% HFIP (Figure S9). Samples were stored for 18 h at 4°C before measurement. Spectra of the corresponding buffer lacking peptide (1% or 2% HFIP/PBS, v/v) were subtracted. For CD melting experiments, Palm1–15 was dissolved at 46 µM in 1% β-OG (w/v) (> critical micelle concentration (CMC) = 0.7% (w/v)) at pH 11.5 and then neutralized to a final concentration of 23 µM in 0.5% β-OG (w/v) (< CMC) at pH 7.4 as previously described [17]. CD spectra were acquired (2 scans) at each temperature from 25°C to 95°C in steps of 5°C and 2°C/min temperature slope (Figure S4). Temperatures equal or lower than 20°C were not included as sample precipitation was observed during measurement that increased the level of noise due to light scattering. Melting temperature (Tm) curves were plotted showing the change in ellipticity (mdeg) at 221 nm and at 205 nm with the temperature (Figure S4). Data were fitted with a sixth order polynomial function, with R^2^ = 0.9776 for 221 nm data and R^2^ = 0.9972 for 205 nm data. Melting temperature was roughly calculated from the maximum of the first derivative of the fitting curve.

### ThT fluorescence

Peptides were prepared as for CD measurement with no incubation time and in triplicate. ThT experiments were done only at 1% HFIP/PBS (v/v), and not at 2% (v/v), as the fluorescence signal/noise ratio was optimal already at 1% HFIP (v/v), compared with negative control (solvent). ThT was added at room temperature to a final concentration of 24 µM. After 30 min, fluorescence emission at 485 nm was measured on a Tecan M200 spectrofluorimeter at 25°C with an excitation of 440 nm. Fluorescence intensity values for each peptide were averaged and normalized to the Palm1–15 emission, taken as 100%.

### ssNMR spectroscopy

NMR experiments were conducted using 3.2 mm and 1.3 mm triple-resonance (^1^H, ^13^C, ^15^N) MAS probes (BrukerBiospin, Germany) at a static magnetic field of 16.4 T corresponding to 700 MHz proton resonance frequency. Adamantane was used as an external reference to calibrate ^13^C and ^1^H resonances under similar experimental conditions (same MAS rate and temperature) [36]. The upfield ^13^C resonance and isotropic ^1^H resonance of adamantane were set to 31.47 and 1.7 ppm, respectively. NMR spectra were processed using Topspin 3.0 (Bruker Biospin) and analyzed with Sparky (Goddard TD, Kneller DG SPARKY 3, University of California, San Francisco, http://www.cgl.ucsf.edu/home/sparky). For Palm1–15 analysis, the amino acids Ala2, Ser8 and Gly9 of Palm1–15 were uniformly labeled with ^13^C and ^15^N and the lyophilized crude powder was used without further treatment. Two-dimensional (^1^H, ^13^C) correlation experiments were conducted using frequency-switched homonuclear Lee-Goldburg [37] decoupling at 83.3 kHz ^1^H field strength during the indirect ^1^H evolution period and regular (^1^H, ^13^C) HETCOR spectra were used for the calibration of the proton dimension. Effective sample temperatures were calibrated using nickelocene. For Palm1–15, two-dimensional (^1^H, ^13^C) and (^13^C, ^13^C) experiments were performed at 0°C at MAS rate of 12 kHz. The signal patterns were compared to known predictions for β-sheet, random-coil or α-helical peptide conformations.

### Attenuated Total Reflectance InfraRed

Spectra were obtained with a BRUKER TENSOR 27 FTIR spectrometer equipped with a liquid nitrogen cooled mercury-cadmium-telluride detector and coupled to a BioATR-II device. For each spectrum 1000 scans were collected with a resolution of 4 cm^−1^ and averaged. The interferograms were recorded double-sided (forward-backward) at a mirror frequency of 20 kHz. The upper and the lower frequency folding limits were 4000 cm^−1^ and 900 cm^−1^. The spectra were FT processed by apodization (Blackman-Harris 3-term function) and double zero filling. The sample chamber was continuously purged with dried air and all measurements were performed at 23°C. Samples were prepared as a film on the ZnSe crystal. Before sample preparation, the trifluoroacetate (CF_3_COO^−^) counterions, which strongly associate with the peptides, were replaced with chloride ions to eliminate the strong absorption band near 1673 cm^−1^. For that purpose, peptide lyophilates were dissolved in a mixture of HFIP and 0.1% HCl_(aq)_ and dried with a speed-vac. Then lipopeptide aggregates were prepared as for CD measurements at 30 µM peptide in 2% HFIP/PBS (v/v). Then, 15 µL of the peptide solution were deposited on the ATR crystal and dried. Spectra were recorded using as background the spectra of the solvent alone. For curve fitting procedure and spectra results, see Method S2 and Figure S8.

### Electron Microscopy

Peptide samples were prepared following the same protocol as for CD studies. After incubation overnight at room temperature, 15 µl sample aliquots were applied to glow discharged carbon-coated grids, washed with distilled water and stained with 15 µl of 1% Uranyl Acetate for 30 s. The excess of stain was removed by blotting with filter paper and grids were dried at room temperature. Visualization was made using a transmission electron microscope Philips CM12 and a Philips CM10 with a voltage of 100 kV at magnification of x 22000. The images were recorded on camera CCD 1024×1024 pixels controlled by the software Digital Micrograph (Gatan) in the CM12 microscope and a Morada camera 4000×2000 pixels in the CM10 microscope. A rough estimation of the fiber size was made by choosing proteins having intact structure. High magnification did not help to recognize single fibers which tended to stay aggregated to each other. The measurements were made on the fibers in which the extremities were clearly visible and without important curvatures (Figure S10). The length of the fibers proteins was measured on 6 pictures using Photoshop tools. The thickness of the fiber was measured similarly along single fibers, from 3 pictures (5 regions). In summary, the average fiber length and average thickness were obtained from several measurements of isolated fibers considering 1 pixel = ∼0.55 nm (Table S2 and Table S3).

## Supporting Information

Figure S1 Absorbance ATR-IR spectra of Palm1–15 (black) and control Acetyl1–15 (grey). The second derivative of the absorbance spectra is plotted, after vector-normalization for comparison.
(TIF)

Figure S2 Electron micrographs of negative stained Acetyl1–15 (top), Aβ1–15 (bottom left) and Palm1–5(4C) (bottom right) at 30 µM in 2% HFIP/PBS (v/v) over 24 hour incubation. Samples were negatively stained with 2% Uranyl Acetate in water. White dots are probably spherical HFIP microdroplets, with some adsorbed peptide [25]. Scale bar 0.1 µm and 50 nm. Magnification, 22000x.
(PNG)

Figure S3 Dynamic Light Scattering (DLS) analysis of Palm1–15 at 50 µg/mL (15 µM) in 1% HFIP**/**PBS solution. Insert: Plot of hydrodynamic diameter of Palm1–15 in 1% HFIP (v/v) at different concentrations of Palm1–15, following serial dilution from 50 µg/mL to 0.25 µg/mL. Values are average of three readings ± standard deviation (error bars are smaller than symbols).
(TIF)

Figure S4 CD Melting temperature experiment of Palm1–15 aggregates in β-OG/PBS instead of HFIP, due to its volatility. a) CD of Palm1–15 aggregates at 23 µM in 0.5% beta-OG/PBS (w/v) pH 7.5, from 25°C to 95°C in steps of 5°C. b) Melting temperature curves monitoring the change in ellipticity (mdeg) at 221 nm and at 205 nm. Melting temperature (∼64°C) was roughly calculated from the maximum of the first derivative of the fitting curve. Data was fitted with a sixth order polynomial function, with R^2^ = 0.9776 for 221 nm data and R^2^ = 0.9972 for 205 nm data. The pre- and post-transition regions in the Tm curve were not totally linear precluding the fitting of these baselines for determining the θF (ellipticity of the fully folded form) and θU (ellipticity of the unfolded form). The calculation of the fraction folded from the ellipticity at 20°C and 100°C, although a very rough approximate, gave similar transition temperature than the one calculated from the maximum of the first derivative of the fitting curve (data not shown).
(TIF)

Figure S5 CD spectra of tetraacetylated version of peptides with shortened length of 9 or 5 amino acids (Acetyl1–9 and Acetyl1–5) or different order of amino acids (reverse or scrambled) with respect to model sequence Palm1–15 (Acetyl15-1 and scAcetyl15); peptides were 30 µM in 2% HFIP/PBS (v/v).
(TIF)

Figure S6 a) CD spectra of control tetraacetylated peptide of mutated sequences with different net charges (pI): Acetyl1–15(E3K, D7K, E11K) (10.0), Acetyl1–15(E3K, D7K) (8.5), Acetyl1–15(A3K, D7K) (7.0) and Acetyl 1–15(D7K) (6.3). Peptides were 30 µM in 2% HFIP/PBS (v/v). b) Thioflavin T fluorescence assay with of tetraacetylated mutated peptides 15 µM in 1% HFIP/PBS (v/v) in the presence of ThT 24 µM. Fluorescence was measured at 485 nm with excitation at 440 nm. Values are the average of 3 replicates, normalized on the Palm1–15 emission (taken as 100%).
(TIF)

Figure S7 a) CD spectra of acetylated peptide controls for the different lipidation patterns: Acetyl1–15(4C) at 40 µM, Acetyl1–15(1N1C) at 47 µM, Acetyl1–15(2C) at 30 µM and Acetyl1–15(1C) at 30 µM, all in 2% HFIP/PBS (v/v). b) ThT fluorescence assay of control peptides acylated with different number/position of acetyl chains; peptides were 15 µM in 1% HFIP/PBS (v/v) in the presence of ThT 24 µM. Fluorescence was measured at 485 nm with excitation at 440 nm. Values are the average of 3 replicates, normalized on the Palm1–15 emission (taken as 100%).
(TIF)

Figure S8 Deconvolution (a–f) of the ATR-IR amide I spectra (g) of the peptides with different lipidation pattern. The components peaks (dotted lines) are the result of curve fitting with PeakFit software. The sums of the fitted components (red line) superimpose on the experimental amide I region cut spectra.
(TIF)

Figure S9 A) CD spectra of tetrapalmitoylated peptides with shortened length, 9 or 5 amino acids (Palm1–9 in blue and Palm1–5 in orange respectively) or different order of amino acids, reverse or scrambled (Palm15-1 in green and scPalm15 in red, respectively) to model sequence Palm1–15. B) CD of tetrapalmitoylated peptides with different isoelectric point (Palm1–15(D7K) in green, Palm1–15(E3A, D7K) in blue, Palm1–15(E3K, D7K) in orange, Palm1–15(E3K, D7K, E11K) in red and Palm1–15 in black). C) CD of peptides with different number/position of palmitic chains (Palm1–15(1C) in orange, Palm1–15(2C) in red, Palm1–15(1N1C) in green, Palm1–15(4C) in blue and Palm1–15 in black). D) CD of peptides acylated with different lipid chain length (Acetyl1–15 in orange, Butyl1–15 in blue, Octyl1–15 in green, Dodecyl1–15 in red and Palm1–15 in black). Peptides were 15 µM in 1% HFIP/PBS (v/v).
(TIF)

Figure S10 Example of measurement of fiber length in EM image (Image D, Table S2). The value is given in pixels, where in this case 166 px = 91 nm (image taken with Camera Morada, 990 px = 550 nm).
(TIF)

Table S1 ssNMR secondary chemical shifts analysis for the ^13^C resonances of ^13^C/^15^N uniformly labeled residues Ala2, Ser8 and Gly9 in Palm1–15.
(PNG)

Table S2 Palm1–15 fiber length distribution from EM images (see representative Figure S10).
(PNG)

Table S3 Palm1–15 fiber thickness distribution from EM images.
(PNG)

Method S1 Dynamic Light Scattering.
(PNG)

Method S2 Curve fitting procedure.
(PNG)

## References

[1] Forns P, Lauer-Fields JL, Gao S, Fields GB (2000) Induction of protein-like molecular architecture by monoalkyl hydrocarbon chains. Biopolymers 54: 531–546. 10984405 10.1002/1097-0282(200012)54:7<531::AID-BIP60>3.0.CO;2-X

[2] Krug M, Folkers G, Haas B, Hess G, Wiesmuller KH, et al (1989) Molecular dynamics of the alpha-helical epitope of a novel synthetic lipopeptide foot-and-mouth disease virus vaccine. Biopolymers 28: 499–512. 2470437 10.1002/bip.360280144

[3] Yu Y-C, Tirrell M, Fields GB (1998) Minimal Lipidation Stabilizes Protein-Like Molecular Architecture. J Am Chem Soc 120: 9979–9987.

[4] Paramonov SE, Jun HW, Hartgerink JD (2006) Self-assembly of peptide-amphiphile nanofibers: the roles of hydrogen bonding and amphiphilic packing. J Am Chem Soc 128: 7291–7298. 16734483 10.1021/ja060573x

[5] Cavalli S, Handgraaf JW, Tellers EE, Popescu DC, Overhand M, et al (2006) Two-dimensional ordered beta-sheet lipopeptide monolayers. J Am Chem Soc 128: 13959–13966. 17044724 10.1021/ja065479v

[6] Lowik DW, Linhardt JG, Adams PJ, van Hest JC (2003) Non-covalent stabilization of a beta-hairpin peptide into liposomes. Org Biomol Chem 1: 1827–1829. 12945757 10.1039/b303749e

[7] Pallavi B, Nagaraj R (2003) Palmitoylated peptides from the cysteine-rich domain of SNAP-23 cause membrane fusion depending on peptide length, position of cysteines, and extent of palmitoylation. J Biol Chem 278: 12737–12744. 12551899 10.1074/jbc.M208598200

[8] Hardy J, Selkoe DJ (2002) The amyloid hypothesis of Alzheimer’s disease: progress and problems on the road to therapeutics. Science 297: 353–356. 12130773 10.1126/science.1072994

[9] Kim W, Hecht MH (2005) Sequence determinants of enhanced amyloidogenicity of Alzheimer A{beta}42 peptide relative to A{beta}40. J Biol Chem 280: 35069–35076. 16079141 10.1074/jbc.M505763200

[10] Burkoth TS, Benzinger TLS, Urban U, Morgan DM, Gregory DM, et al (2000) Structure of the ß-Amyloid(10-35) Fibril. J Am Chem Soc 122: 7883–7889 doi:10.1021/ja000645z

[11] Gordon DJ, Balbach JJ, Tycko R, Meredith SC (2004) Increasing the amphiphilicity of an amyloidogenic peptide changes the beta-sheet structure in the fibrils from antiparallel to parallel. Biophys J 86: 428–434. 14695285 10.1016/S0006-3495(04)74119-3 PMC1303808

[12] Petkova AT, Ishii Y, Balbach JJ, Antzutkin ON, Leapman RD, et al (2002) A structural model for Alzheimer’s beta-amyloid fibrils based on experimental constraints from solid state NMR. Proc Natl Acad Sci U S A 99: 16742–16747. 12481027 10.1073/pnas.262663499 PMC139214

[13] Qahwash IM, Boire A, Lanning J, Krausz T, Pytel P, et al (2007) Site-specific effects of peptide lipidation on beta-amyloid aggregation and cytotoxicity. J Biol Chem 282: 36987–36997. 17693400 10.1074/jbc.M702146200

[14] Tycko R (2003) Insights into the amyloid folding problem from solid-state NMR. Biochemistry 42: 3151–3159. 12641446 10.1021/bi027378p

[15] Luhrs T, Ritter C, Adrian M, Riek-Loher D, Bohrmann B, et al (2005) 3D structure of Alzheimer’s amyloid-beta(1–42) fibrils. Proc Natl Acad Sci U S A 102: 17342–17347. 16293696 10.1073/pnas.0506723102 PMC1297669

[16] Olofsson A, Sauer-Eriksson AE, Ohman A (2006) The solvent protection of Alzheimer amyloid-beta-(1–42) fibrils as determined by solution NMR spectroscopy. J Biol Chem 281: 477–483. 16215229 10.1074/jbc.M508962200

[17] Hickman DT, Lopez-Deber MP, Ndao DM, Silva AB, Nand D, et al (2011) Sequence-independent Control of Peptide Conformation in Liposomal Vaccines for Targeting Protein Misfolding Diseases. J Biol Chem 286: 13966–13976. 21343310 10.1074/jbc.M110.186338 PMC3077597

[18] Koers EJ, Lopez-Deber MP, Weingarth M, Nand D, Hickman DT, et al (2013) Dynamic Nuclear Polarization NMR Spectroscopy: Revealing Multiple Conformations in Lipid-Anchored Peptide Vaccines. Angew Chem Int Ed Engl 52: 10905–10908. 24039018 10.1002/anie.201303374

[19] Cerf E, Sarroukh R, Tamamizu-Kato S, Breydo L, Derclaye S, et al (2009) Antiparallel beta-sheet: a signature structure of the oligomeric amyloid beta-peptide. Biochem J 421: 415–423. 19435461 10.1042/BJ20090379

[20] Serpell LC (2000) Alzheimer’s amyloid fibrils: structure and assembly. Biochim Biophys Acta 1502: 16–30. 10899428 10.1016/s0925-4439(00)00029-6

[21] Lomakin A, Chung DS, Benedek GB, Kirschner DA, Teplow DB (1996) On the nucleation and growth of amyloid beta-protein fibrils: detection of nuclei and quantitation of rate constants. Proc Natl Acad Sci U S A 93: 1125–1129. 8577726 10.1073/pnas.93.3.1125 PMC40042

[22] Buck M (1998) Trifluoroethanol and colleagues: cosolvents come of age. Recent studies with peptides and proteins. Q Rev Biophys 31: 297–355. 10384688 10.1017/s003358359800345x

[23] Zagorski MG, Yang J, Shao H, Ma K, Zeng H, et al (1999) Methodological and chemical factors affecting amyloid beta peptide amyloidogenicity. Methods Enzymol 309: 189–204. 10507025 10.1016/s0076-6879(99)09015-1

[24] Roccatano D, Fioroni M, Zacharias M, Colombo G (2005) Effect of hexafluoroisopropanol alcohol on the structure of melittin: a molecular dynamics simulation study. Protein Sci 14: 2582–2589. 16155200 10.1110/ps.051426605 PMC2253304

[25] Nichols MR, Moss MA, Reed DK, Hoh JH, Rosenberry TL (2005) Amyloid-beta aggregates formed at polar-nonpolar interfaces differ from amyloid-beta protofibrils produced in aqueous buffers. Microsc Res Tech 67: 164–174. 16103999 10.1002/jemt.20189

[26] Nichols MR, Moss MA, Reed DK, Cratic-McDaniel S, Hoh JH, et al (2005) Amyloid-beta protofibrils differ from amyloid-beta aggregates induced in dilute hexafluoroisopropanol in stability and morphology. J Biol Chem 280: 2471–2480. 15528204 10.1074/jbc.M410553200

[27] Padrick SB, Miranker AD (2002) Islet amyloid: phase partitioning and secondary nucleation are central to the mechanism of fibrillogenesis. Biochemistry 41: 4694–4703. 11926832 10.1021/bi0160462

[28] Yanagi K, Ashizaki M, Yagi H, Sakurai K, Lee YH, et al (2011) Hexafluoroisopropanol induces amyloid fibrils of islet amyloid polypeptide by enhancing both hydrophobic and electrostatic interactions. J Biol Chem 286: 23959–23966. 21566116 10.1074/jbc.M111.226688 PMC3129177

[29] Schneider R, Schumacher MC, Mueller H, Nand D, Klaukien V, et al (2011) Structural characterization of polyglutamine fibrils by solid-state NMR spectroscopy. J Mol Biol 412: 121–136. 21763317 10.1016/j.jmb.2011.06.045

[30] Gursky O, Aleshkov S (2000) Temperature-dependent beta-sheet formation in beta-amyloid Abeta(1–40) peptide in water: uncoupling beta-structure folding from aggregation. Biochim Biophys Acta 1476: 93–102. 10606771 10.1016/s0167-4838(99)00228-9

[31] Nichols MR, Moss MA, Reed DK, Hoh JH, Rosenberry TL (2005) Rapid assembly of amyloid-beta peptide at a liquid/liquid interface produces unstable beta-sheet fibers. Biochemistry 44: 165–173. 15628857 10.1021/bi048846t

[32] Rangachari V, Reed DK, Moore BD, Rosenberry TL (2006) Secondary structure and interfacial aggregation of amyloid-beta(1–40) on sodium dodecyl sulfate micelles. Biochemistry 45: 8639–8648. 16834338 10.1021/bi060323t

[33] Epand RM (1997) Biophysical studies of lipopeptide-membrane interactions. Biopolymers 43: 15–24. 9174409 10.1002/(SICI)1097-0282(1997)43:1<15::AID-BIP3>3.0.CO;2-3

[34] Rushworth JV, Hooper NM (2010) Lipid Rafts: Linking Alzheimer’s Amyloid-beta Production, Aggregation, and Toxicity at Neuronal Membranes. Int J Alzheimers Dis 2011: 603052. 21234417 10.4061/2011/603052 PMC3014710

[35] Zhang Q, Powers ET, Nieva J, Huff ME, Dendle MA, et al (2004) Metabolite-initiated protein misfolding may trigger Alzheimer’s disease. Proc Natl Acad Sci U S A 101: 4752–4757. 15034169 10.1073/pnas.0400924101 PMC387320

[36] Morcombe CR, Zilm KW (2003) Chemical shift referencing in MAS solid state NMR. J Magn Reson 162: 479–486. 12810033 10.1016/s1090-7807(03)00082-x

[37] Bielecki A, Kolbert AC, Levitt MH (1989) Frequency-switched pulse sequences: Homonuclear decoupling and dilute spin NMR in solids. Chem Phys Lett 155: 341–346.

[38] Wang Y, Jardetzky O (2002) Probability-based protein secondary structure identification using combined NMR chemical-shift data. Protein Sci 11: 852–861. 11910028 10.1110/ps.3180102 PMC2373532

