# Supplementary figures and images for: Engineering Amyloid-Like Assemblies from Unstructured Peptides via Site-Specific Lipid Conjugation
Source: PLoS One. 2014 Sep 10;9(9):e105641. doi: 10.1371/journal.pone.0105641 (PMC4160191; doi:10.1371/journal.pone.0105641)

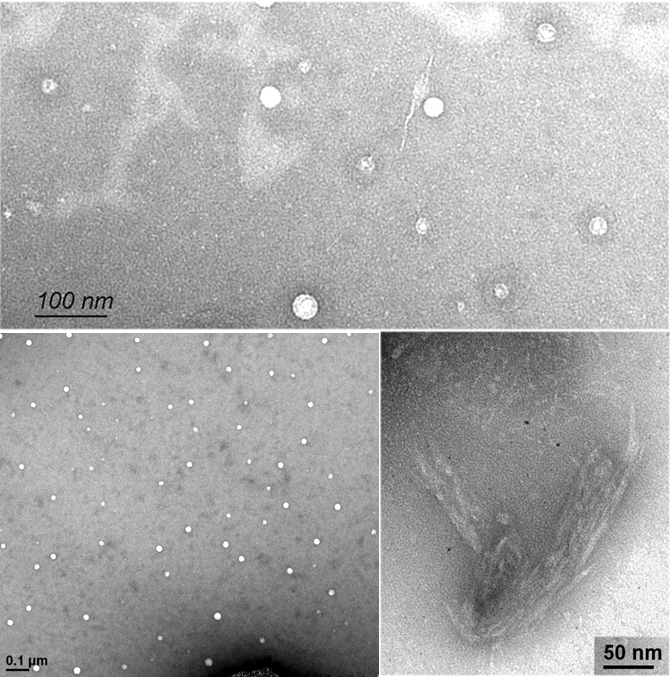

Supplement: Figure S2 — Electron micrographs of negative stained Acetyl1–15 (top), Aβ1–15 (bottom left) and Palm1–5(4C) (bottom right) at 30 µM in 2% HFIP/PBS (v/v) over 24 hour incubation. Samples were negatively stained with 2% Uranyl Acetate in water. White dots are probably spherical HFIP microdroplets, with some adsorbed peptide [25]. Scale bar 0.1 µm and 50 nm. Magnification, 22000x. (PNG) [file pone.0105641.s002.png]

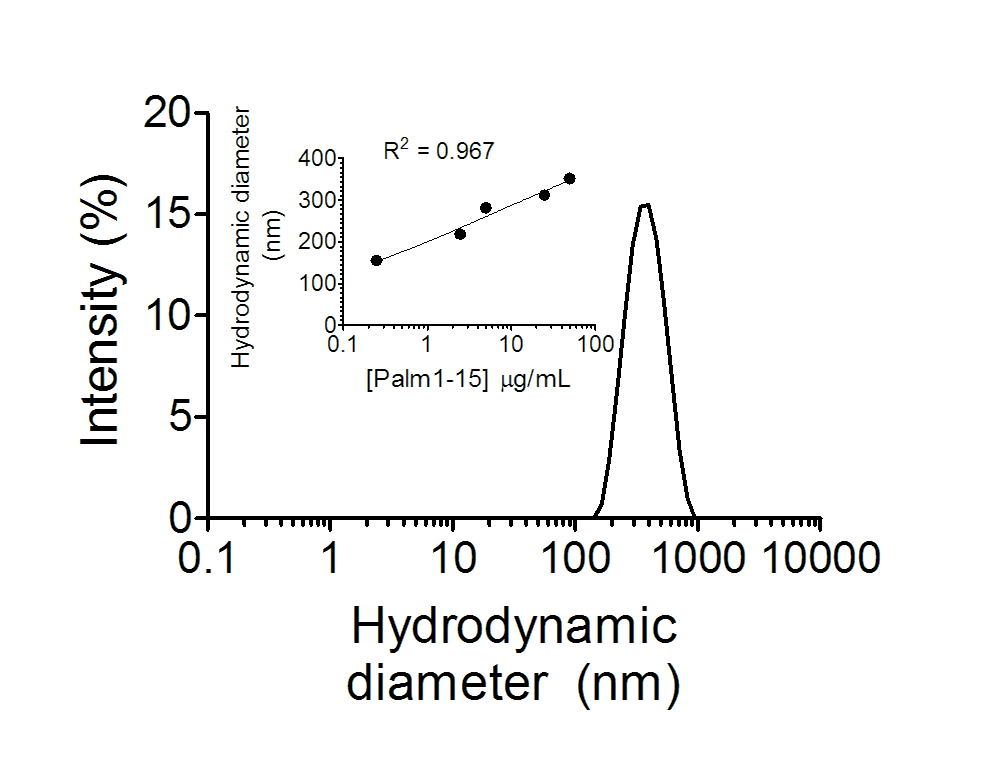

Supplement: Figure S3 — Dynamic Light Scattering (DLS) analysis of Palm1–15 at 50 µg/mL (15 µM) in 1% HFIP/PBS solution. Insert: Plot of hydrodynamic diameter of Palm1–15 in 1% HFIP (v/v) at different concentrations of Palm1–15, following serial dilution from 50 µg/mL to 0.25 µg/mL. Values are average of three readings ± standard deviation (error bars are smaller than symbols). (TIF) [file pone.0105641.s003.tif]

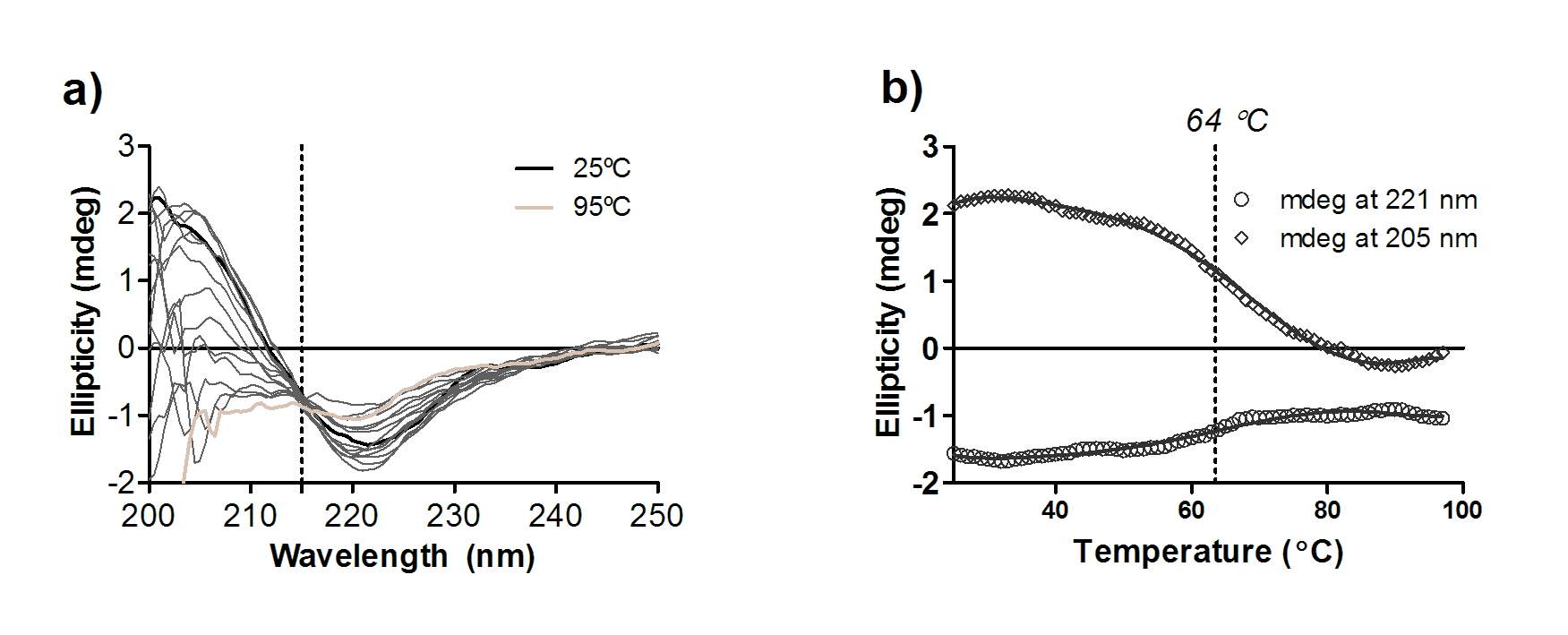

Supplement: Figure S4 — CD Melting temperature experiment of Palm1–15 aggregates in β-OG/PBS instead of HFIP, due to its volatility. a) CD of Palm1–15 aggregates at 23 µM in 0.5% beta-OG/PBS (w/v) pH 7.5, from 25°C to 95°C in steps of 5°C. b) Melting temperature curves monitoring the change in ellipticity (mdeg) at 221 nm and at 205 nm. Melting temperature (∼64°C) was roughly calculated from the maximum of the first derivative of the fitting curve. Data was fitted with a sixth order polynomial function, with R2 = 0.9776 for 221 nm data and R2 = 0.9972 for 205 nm data. The pre- and post-transition regions in the Tm curve were not totally linear precluding the fitting of these baselines for determining the θF (ellipticity of the fully folded form) and θU (ellipticity of the unfolded form). The calculation of the fraction folded from the ellipticity at 20°C and 100°C, although a very rough approximate, gave similar transition temperature than the one calculated from the maximum of the first derivative of the fitting curve (data not shown). (TIF) [file pone.0105641.s004.tif]

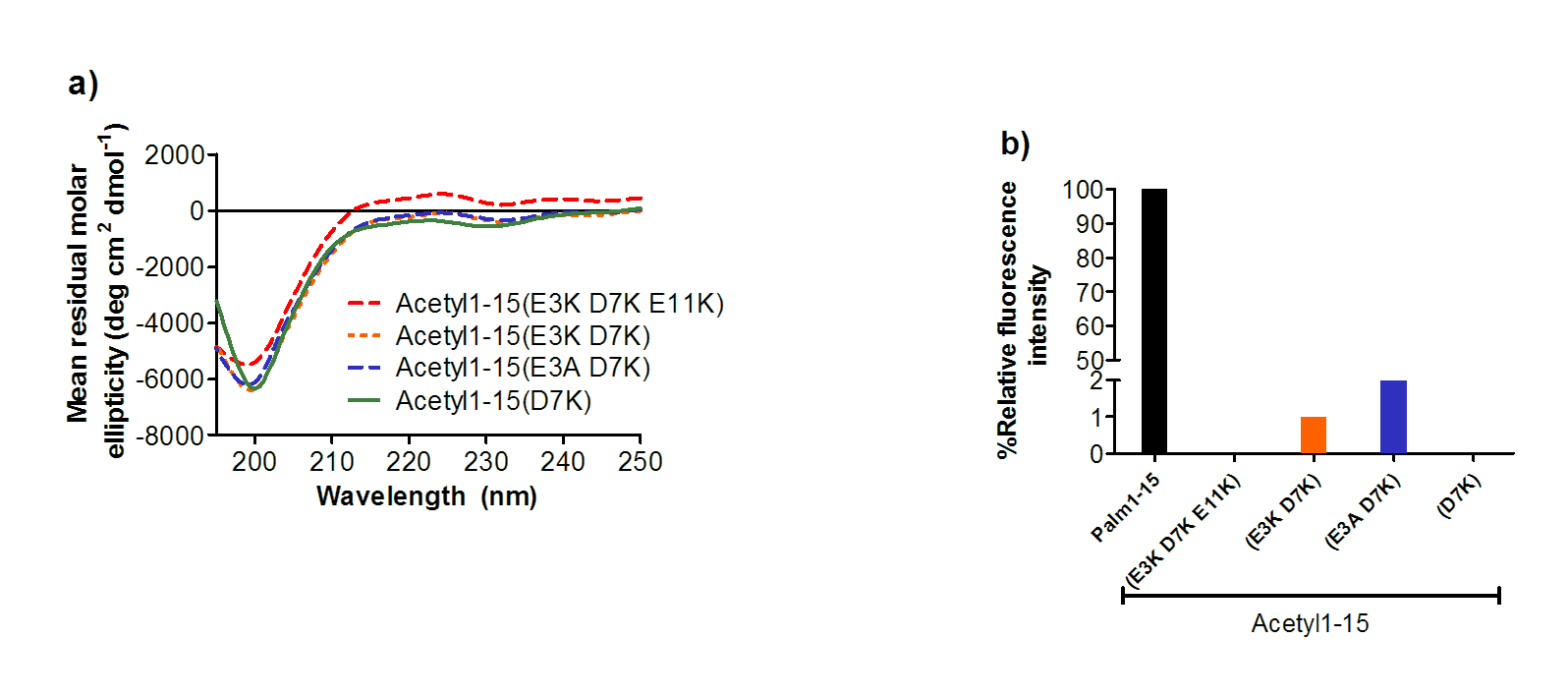

Supplement: Figure S6 — a) CD spectra of control tetraacetylated peptide of mutated sequences with different net charges (pI): Acetyl1–15(E3K, D7K, E11K) (10.0), Acetyl1–15(E3K, D7K) (8.5), Acetyl1–15(A3K, D7K) (7.0) and Acetyl 1–15(D7K) (6.3). Peptides were 30 µM in 2% HFIP/PBS (v/v). b) Thioflavin T fluorescence assay with of tetraacetylated mutated peptides 15 µM in 1% HFIP/PBS (v/v) in the presence of ThT 24 µM. Fluorescence was measured at 485 nm with excitation at 440 nm. Values are the average of 3 replicates, normalized on the Palm1–15 emission (taken as 100%). (TIF) [file pone.0105641.s006.tif]

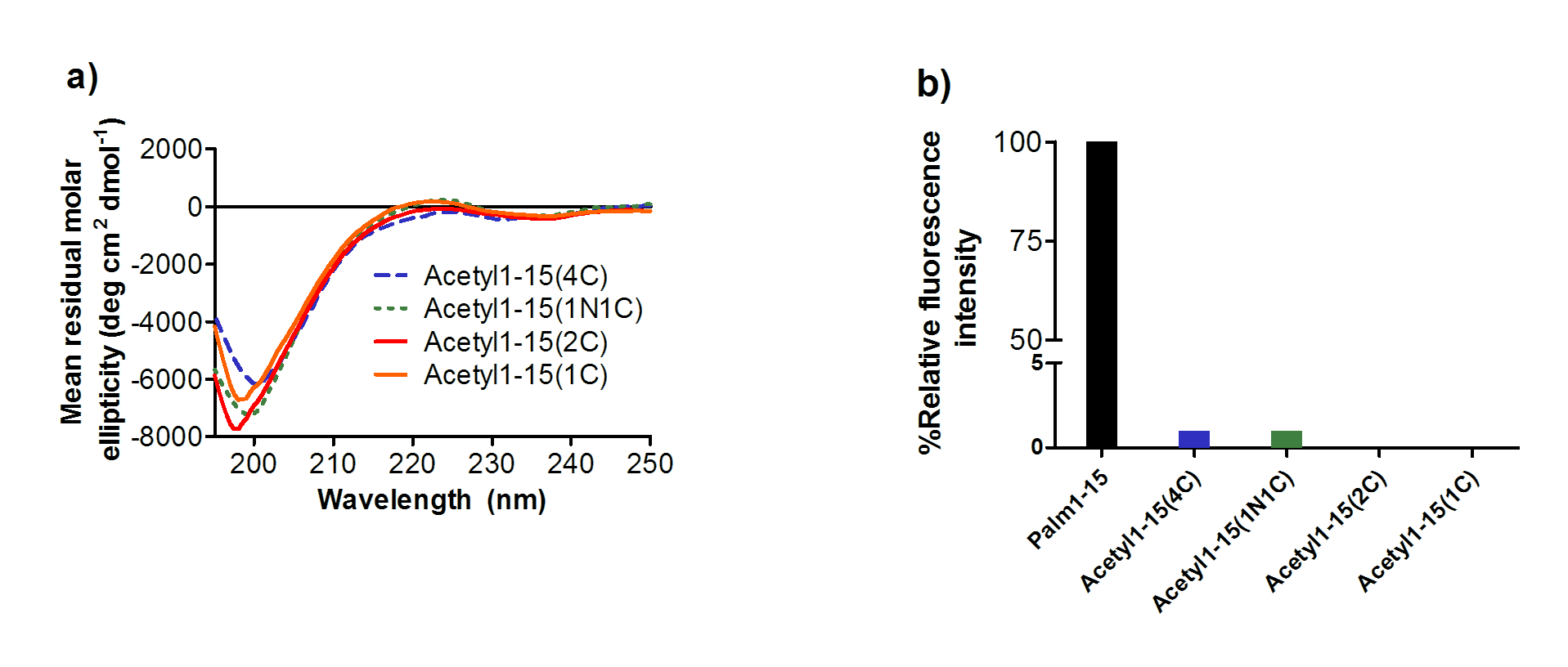

Supplement: Figure S7 — a) CD spectra of acetylated peptide controls for the different lipidation patterns: Acetyl1–15(4C) at 40 µM, Acetyl1–15(1N1C) at 47 µM, Acetyl1–15(2C) at 30 µM and Acetyl1–15(1C) at 30 µM, all in 2% HFIP/PBS (v/v). b) ThT fluorescence assay of control peptides acylated with different number/position of acetyl chains; peptides were 15 µM in 1% HFIP/PBS (v/v) in the presence of ThT 24 µM. Fluorescence was measured at 485 nm with excitation at 440 nm. Values are the average of 3 replicates, normalized on the Palm1–15 emission (taken as 100%). (TIF) [file pone.0105641.s007.tif]

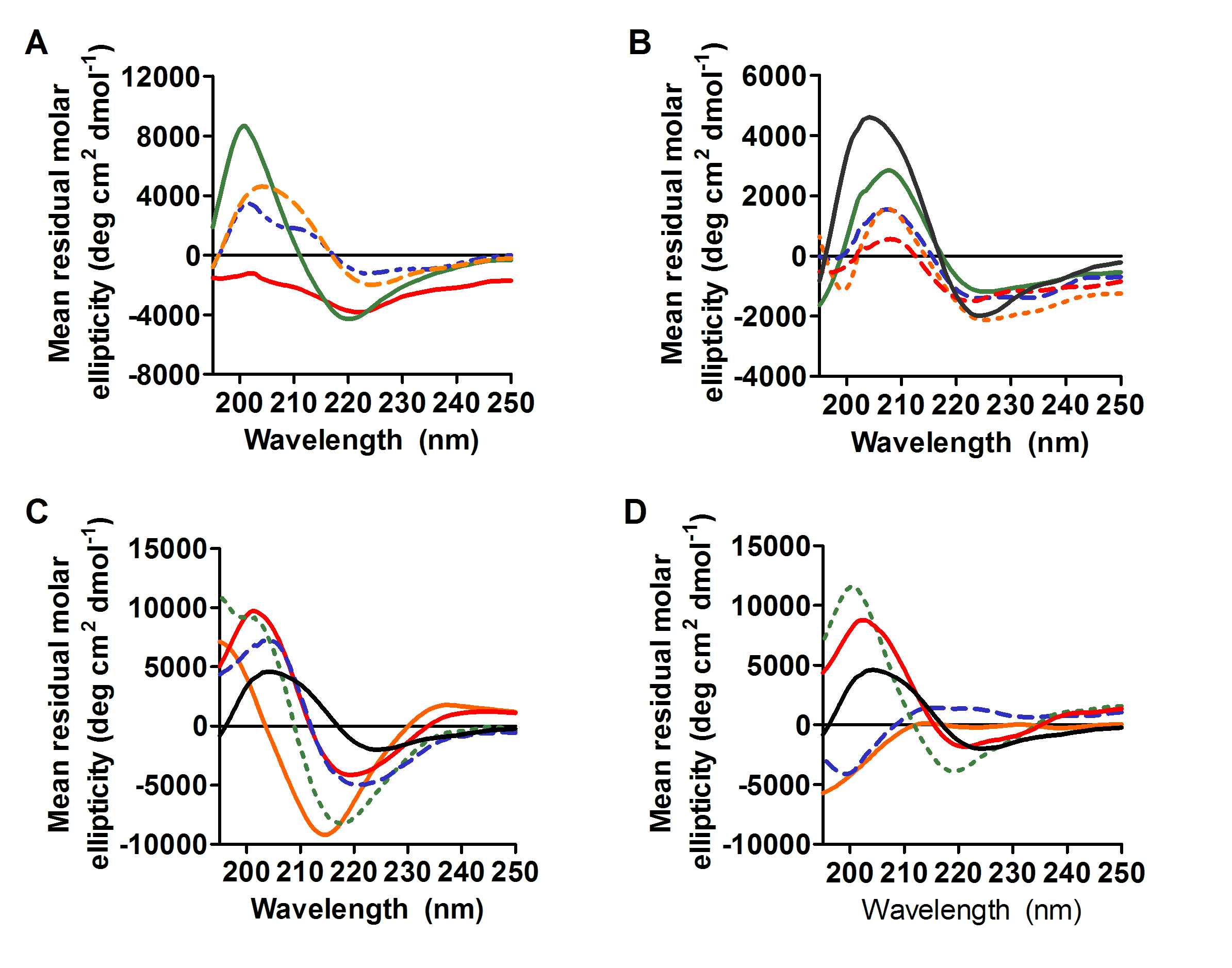

Supplement: Figure S9 — A) CD spectra of tetrapalmitoylated peptides with shortened length, 9 or 5 amino acids (Palm1–9 in blue and Palm1–5 in orange respectively) or different order of amino acids, reverse or scrambled (Palm15-1 in green and scPalm15 in red, respectively) to model sequence Palm1–15. B) CD of tetrapalmitoylated peptides with different isoelectric point (Palm1–15(D7K) in green, Palm1–15(E3A, D7K) in blue, Palm1–15(E3K, D7K) in orange, Palm1–15(E3K, D7K, E11K) in red and Palm1–15 in black). C) CD of peptides with different number/position of palmitic chains (Palm1–15(1C) in orange, Palm1–15(2C) in red, Palm1–15(1N1C) in green, Palm1–15(4C) in blue and Palm1–15 in black). D) CD of peptides acylated with different lipid chain length (Acetyl1–15 in orange, Butyl1–15 in blue, Octyl1–15 in green, Dodecyl1–15 in red and Palm1–15 in black). Peptides were 15 µM in 1% HFIP/PBS (v/v). (TIF) [file pone.0105641.s009.tif]

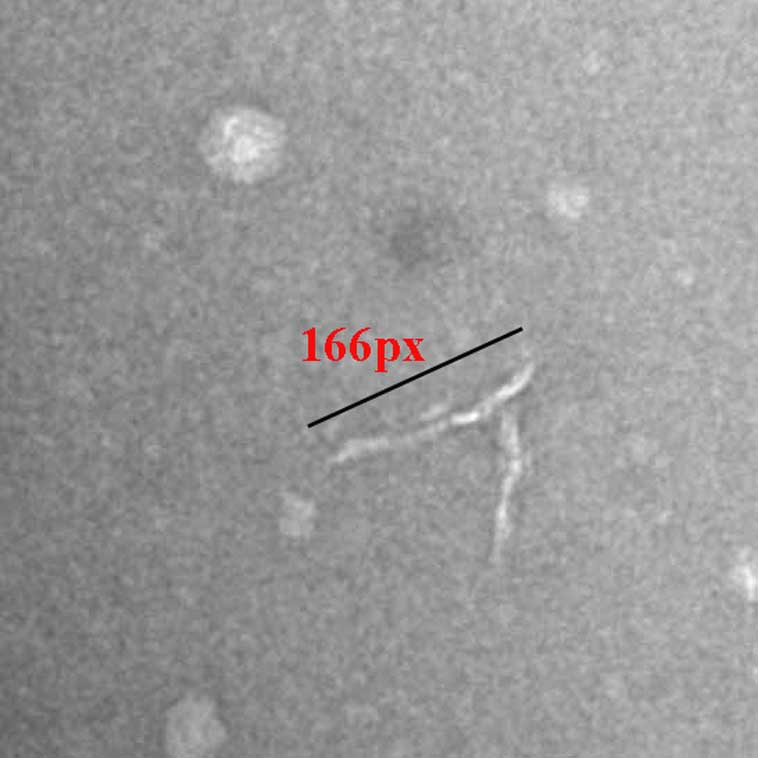

Supplement: Figure S10 — Example of measurement of fiber length in EM image (Image D, Table S2). The value is given in pixels, where in this case 166 px = 91 nm (image taken with Camera Morada, 990 px = 550 nm). (TIF) [file pone.0105641.s010.tif]

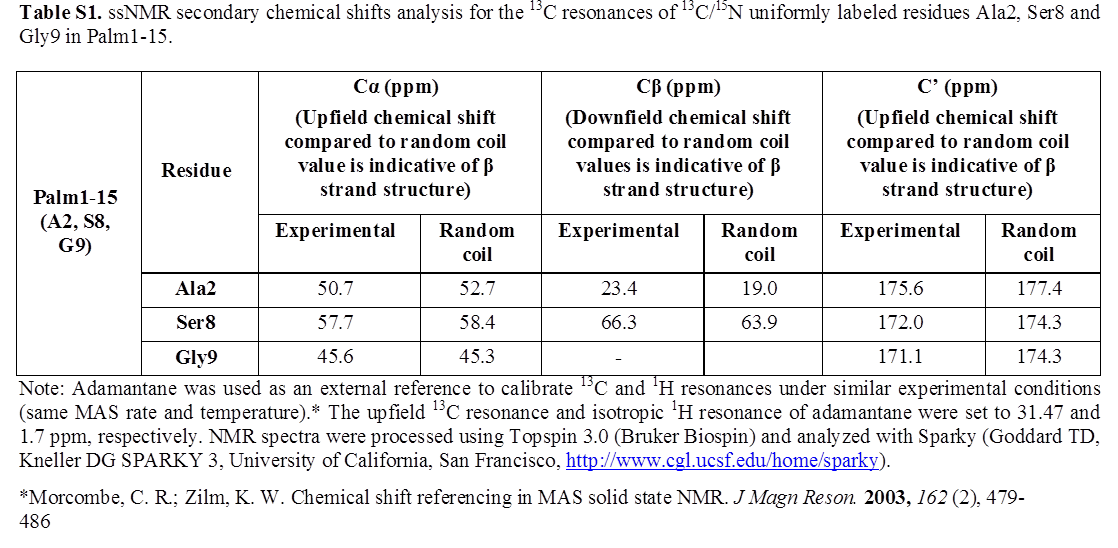

Supplement: Table S1 — ssNMR secondary chemical shifts analysis for the 13C resonances of 13C/15N uniformly labeled residues Ala2, Ser8 and Gly9 in Palm1–15. (PNG) [file pone.0105641.s011.png]

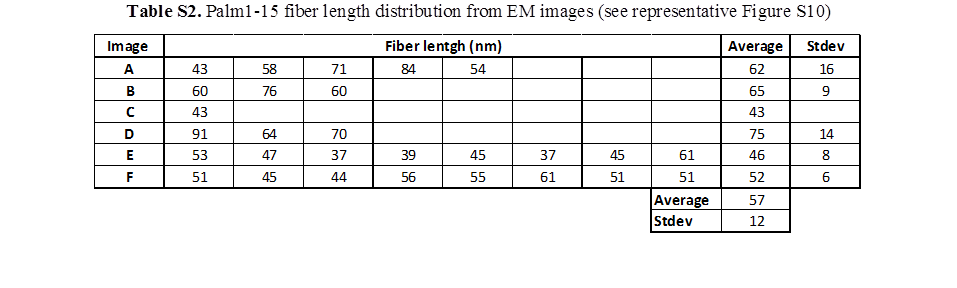

Supplement: Table S2 — Palm1–15 fiber length distribution from EM images (see representative Figure S10). (PNG) [file pone.0105641.s012.png]

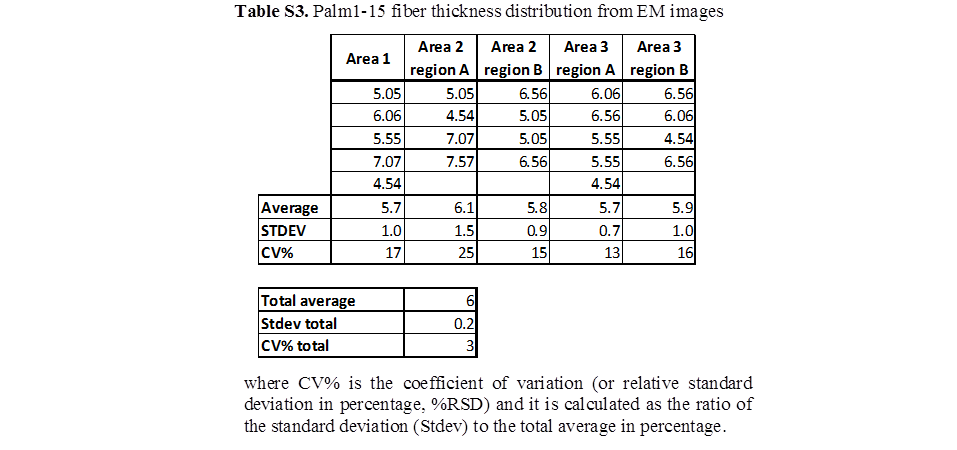

Supplement: Table S3 — Palm1–15 fiber thickness distribution from EM images. (PNG) [file pone.0105641.s013.png]

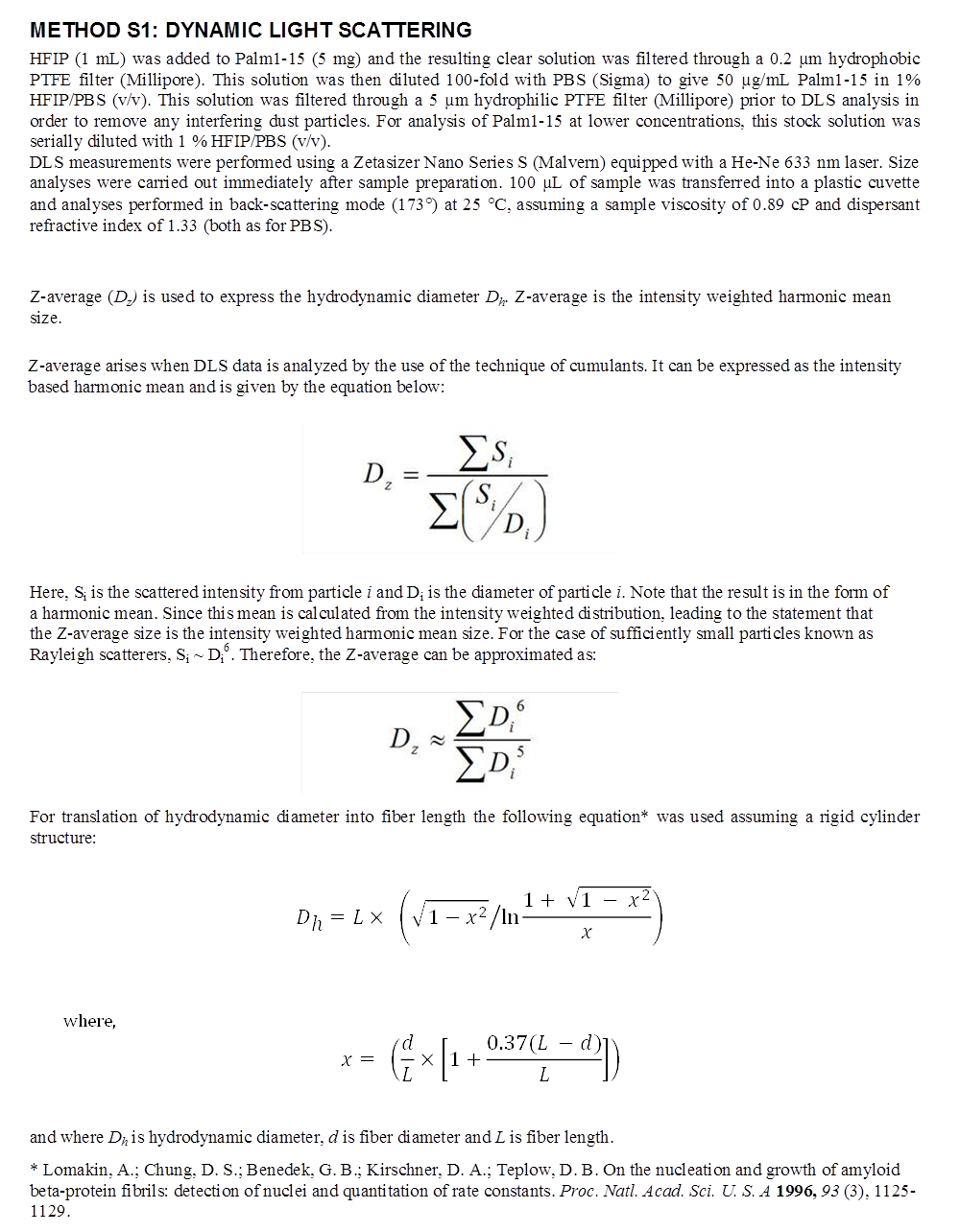

Supplement: Method S1 — Dynamic Light Scattering. (PNG) [file pone.0105641.s014.png]

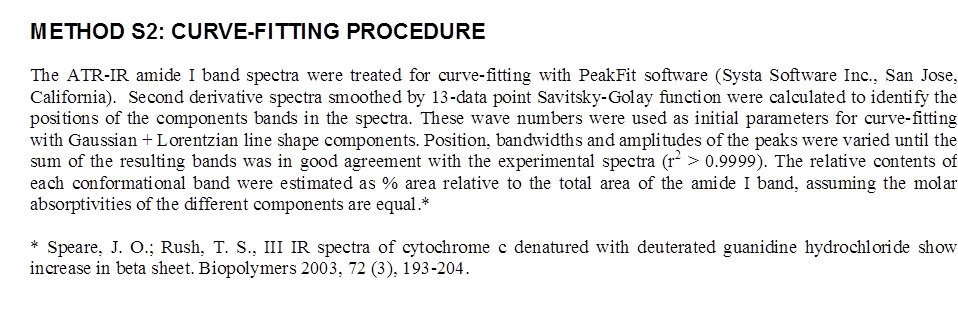

Supplement: Method S2 — Curve fitting procedure. (PNG) [file pone.0105641.s015.png]
